# Curculigoside promotes osteogenic differentiation of ADSCs to prevent ovariectomized-induced osteoporosis

**DOI:** 10.1186/s13018-021-02389-3

**Published:** 2021-04-26

**Authors:** Wei-Li You, Zheng-Long Xu

**Affiliations:** 1 Department of Pharmacy, The First People’s Hospital of Lianyungang, No. 128, Tongguanbei Road, Haizhou District, Lianyungang, 222002 Jiangsu Province China; 2 Department of Pharmacy, Xinghua City People’s Hospital, Xinghua City, Jiangsu Province China

**Keywords:** Adipose-derived stem cells, Curculigoside, PI3K/Akt signaling pathway, Osteoporosis

## Abstract

**Background:**

Curculigoside is a natural phenolic glycoside compound produced by *Curculigo orchioides* Gaertn. This study aimed to explore the effects of curculigoside in promoting the osteogenic differentiation of adipose-derived stem cells (ADSCs) as well as the underlying mechanism.

**Methods:**

ADSCs were treated with curculigoside at different concentrations (0 μmol/L, 1 μmol/L, 2.5 μmol/L, 5 μmol/L, 10 μmol/L, and 20 μmol/L), and cell viability was assessed by CCK-8 assay. Then, the alkaline phosphatase (ALP) activity was determined, and alizarin red S (ARS) staining was performed to measure the extracellular mineralization of curculigoside. Information about protein-chemical interactions is provided by the search tool for interactions of chemicals (STITCH) database. Then, LY294002 was administered to explore the mechanism by which curculigoside promotes the osteogenic differentiation of ADSCs. Western blot assays were performed to assess changes in the expression of osteogenic-related markers and the phosphorylation of PI3K and AKT. Finally, we established an ovariectomized (OVX)-induced osteoporosis mouse model and administered curculigoside to explore the effects of curculigoside in preventing bone loss in vivo.

**Results:**

The CCK-8 assay indicated that curculigoside did not induce cytotoxicity at a concentration of 5 μmol/L after 48 h. The ALP and ARS results revealed that the induced group had higher ALP activity and calcium deposition than the control group. Moreover, the curculigoside group exhibited increased biomineralization, ALP activity, and ARS staining compared to the induced and control groups, and these effects were partially inhibited by LY294002. Kyoto Encyclopedia of Genes and Genomes (KEGG) enrichment analysis indicated that the target genes of curculigoside were mainly involved in the PI3K-Akt signaling pathway. PCR and western blot analysis showed that the expression of RUNX2, ALP, and Osterix was upregulated in curculigoside-treated ADSCs, but this effect was partially reversed by the PI3K inhibitor LY294002. Moreover, the curculigoside-treated group exhibited significantly increased phosphorylation of AKT to P-AKT compared with the osteogenic induction group. After treatment with curculigoside, the mice had a higher bone volume than the OVX mice, suggesting partial protection from cancellous bone loss. In addition, when LY294002 was added, the protective effects of curculigoside could be neutralized.

**Conclusions:**

Curculigoside could induce the osteogenic differentiation of ADSCs and prevent bone loss in an OVX model through the PI3K/Akt signaling pathway.

## Background

Osteoporosis is a systemic bone metabolism disease that is caused by bone loss and bone microstructure damage [1–3]. As the population ages, the prevalence of osteoporosis increases year by year [4]. The pathogenesis of osteoporosis involves the disruption of the dynamic balance between bone resorption and bone formation [5–7]. Bone resorption exceeds bone formation, ultimately leading to bone mass decreases. Bone strength in osteoporosis patients is decreased, and the bones are prone to fracture [8]. Therefore, seeking suitable drugs to promote osteoblastic bone formation or inhibit osteoclast-mediated bone resorption has very important practical significance for the treatment of osteoporosis [9].

At present, among the many drugs used for the treatment of osteoporosis, parathyroid hormone is the only one used to promote bone formation and that is approved by the US Food and Drug Administration (FDA) [10]. However, the application of parathyroid hormone can lead to osteosarcoma, which limits its large-scale clinical application [11–13]. Thus, researchers have begun working to identify natural compounds that may promote bone formation [14]. Curculigoside is a natural phenolic glycoside compound produced by *Curculigo orchioides* Gaertn [15]. The pharmacological effects of curculigoside are quite extensive and include antioxidative, anti-inflammatory, antidepressant, and antiosteoporotic effects [16].

Adipose-derived stem cells (ADSCs) exhibit high potential to differentiate into multilineage cells, including osteoblasts, chondrocytes, adipocytes, and fibroblasts [17–19]. A theoretical inverse relationship exists between the osteogenic and adipogenic differentiation of ADSCs [20]. The specific inhibition of ADSC adipogenesis and a concomitant enhancement of ADSC osteogenesis may provide a novel therapeutic approach for the treatment of osteoporosis [21].

The PI3K/Akt signal transduction pathway is the downstream signal transduction pathway mediated by the PI3K family, and this pathway plays an important role in regulating cell differentiation [22, 23]. Akt is a downstream target of PI3K and is also a critical mediator of survival signals for the promotion of cell differentiation [24]. The PI3K-Akt signaling pathway regulates many normal cellular processes, and aberrant activation of the PI3K-Akt pathway has been widely implicated in osteogenic differentiation [25]. However, whether curculigoside plays a beneficial role in promoting the osteogenic differentiation of ADSCs is unknown.

In this study, we assessed the role of curculigoside in promoting the osteogenic differentiation of ADSCs, as well as the underlying mechanism, through in vivo and in vitro studies.

## Methods

### Chemicals and reagents

Curculigoside (chemical purity = 99.73%) was purchased from MCE company (CAS: 85643-19-2, cat: HY-N0705, MedChemExpress, NJ, USA). Dulbecco’s modified Eagle’s medium (DMEM) and fetal bovine serum (FBS) were purchased from Gibco (Life Technologies, Shanghai, China). The ALP activity kit and ALP staining kit were purchased from Beyotime Company (Shanghai, China). Alizarin Red S was purchased from Solarbio Company (Beijing, China).

### ADSC isolation and culture

Human subcutaneous adipose tissue was obtained using liposuction. The adipose tissue was cut into small pieces and digested with 0.075% type I collagenase and trypsin in a shaking water bath at 37 °C for 30 min. Then, L-DMEM culture medium containing 10% FBS was added and filtered with a 100-μm cell sieve to remove the undigested tissue mass. The cell suspension was centrifuged at 1500 rpm to remove the supernatant and resuspended in 5 mL of DMEM + 20% FBS. When the cultured primary cells reached 70–80% confluence, they were subcultured and used for further studies. Cells at passages three to five were used for experiments.

### CCK-8 assays

ADSCs (1 × 10^3^ per well) were plated in a 96-well plate and then treated with basal medium or basal medium containing curculigoside at concentrations of 1, 2.5, 5, 10, and 20 μmol/L. After 1, 3, and 7 days, cell proliferation was determined by the CCK-8 assay (Solarbio, Beijing, China) according to the manufacturer’s instructions. After washing with 1x PBS, 10 μL CCK-8 reagent plus 100 μL basal DMEM medium was added per well and incubated at 37 °C for 2 h. The absorbance at 450 nm was measured with a microplate reader (Multiskan FC, Thermo Scientific, Shanghai, China). The optical density (OD) values were measured, and the cell proliferation rate was calculated.

### Osteogenic differentiation and cell treatments

For osteogenic differentiation, ADSCs were incubated at 1×10^4^ cells/cm^2^ (2 mL/well) in a 6-well plate. When the cell confluence reached 80%, the ADSCs were treated with osteogenic induction medium containing 0, 1, 2.5, and 5 μmol/L curculigoside. Osteogenic induction medium mainly included DMEM supplemented with 10 nM dexamethasone, 50 mug ml-1 ascorbic acid, and 0.1 mM beta-glycerophosphate.

### ALP activity determination

Intracellular ALP activity was measured using a colorimetric ALP assay (Sigma Aldrich) as described previously at all four indicated timepoints and under all indicated conditions. Then, the absorbance of the solution at 450 nm was measured using a microplate reader (Multiskan FC, Thermo Fisher Scientific, Shanghai, China).The results for ALP activity were normalized to the level of the total protein as determined by the Bradford method.

### Alizarin Red S staining

After 21 days, the cells were fixed in 4% paraformaldehyde at room temperature for 30 min. Then, ADSCs were washed twice with PBS and stained with Alizarin Red S (0.2%, pH 8.3) for 30 min at room temperature. After washing with distilled deionized water, the ADSCs were observed and photographed under an optical microscope (Leica DM 500, Leica light microscope, Wetzlar, Germany). To quantify mineralization, calcium deposits were desorbed using 10% cetylpyridinium chloride (Sigma-Aldrich; Merck KGaA), and the absorbance at 570 nm was measured in triplicate plates using a microtiter plate reader.

### Real-time quantitative PCR

Total RNA was extracted from ADSCs using TRIzol (Invitrogen) following the manufacturer’s protocol. cDNA was synthesized using the cDNA Reverse Transcription kit (TAKARA) according to the manufacturer’s instructions. RT-qPCR analyses were conducted by using SYBR Green PCR master mix (Takara, Tokyo, Japan) in the Light Cycler 480 Real-time PCR system (Roche Molecular Systems, Basel, Switzerland). The multiple change was calculated by 2^−△△Ct^ method.

### Western blotting assays

After the indicated treatment, ADSCs were washed twice with prechilled PBS buffer. The harvested ADSCs were lysed on ice for 30 min in RIPA lysis buffer (Beyotime Biotechnology, Shanghai, China) containing 100 mM PMSF (Beyotime Biotechnology) to extract total proteins. The protein concentrations were measured by the BCA protein assay kit (Solarbio, Beijing, China). Then, equal amounts of protein extracts (25 μg) were subjected to 10% SDS-PAGE as described. Next, the proteins were transferred electrophoretically to polyvinylidene difluoride (PVDF) membranes (Millipore Corporation, Billerica, MA, USA).

After blocking with 5% skim milk for 2 h at room temperature, the membranes were incubated with specific primary antibodies against RUNX2, ALP, and Osterix (Santa Cruz, CA, USA) overnight at 4 °C. The membranes were then washed three times with TBS-Tween 20 and incubated with HRP-conjugated secondary antibodies for 1 h at room temperature. Immunoreactive bands were visualized with ECL plus (GE Life Sciences) according to the manufacturer’s instructions. The protein band densities were analyzed using ImageJ software (version 18.0, National Institutes of Health, Bethesda, MD, USA).

### OVX-induced osteoporosis mouse model establishment and curculigoside treatment

All the experiments were performed according to protocols approved by the Institutional Animal Care Committee of The First People’s Hospital of Lianyungang. Eight-week-old female C57BL/6 mice were purchased from the Beijing Animal Institute (Beijing, China) and randomly divided into four groups (Sham group, OVX group, OVX + Curculigoside group, and OVX + Curculigoside +LY294002 group, *n* = 8). Curculigoside was dissolved in dimethylsulfoxide (DMSO) and diluted in PBS.

Bilateral ovariectomy was performed to establish an OVX mouse model of osteoporosis. In the sham procedure, animals were anesthetized, and the ovaries were not removed. For the OVX + curculigoside group, curculigoside was administered via an intraperitoneal injection of 100 μl curculigoside (7.5 mg/kg body weight) for 4 weeks. For LY294002 administration, the mice were intraperitoneally injected with LY294002 (4~5 g per gram body weight) 1 week after curculigoside treatment.

### Histological and immunohistochemical analyses

After fixation of the distal femur in 4% paraformaldehyde, the femur was decalcified in 0.5 M EDTA in PBS for 2 months. The EDTA solution was replaced every day until complete decalcification had occurred. The samples were then paraffin-embedded, and 5-μm sections were produced. Then, HE staining was performed with a HE staining kit (Beyotime, China) according to the manufacturer’s instructions.

The paraffin sections were incubated for 1 h at 70 °C, deparaffinized in xylene, and rehydrated in graded ethanol. Then, endogenous peroxidase activity was quenched by 3% H_2_O_2_ for 10 min. The sections were deparaffinized, and antigen retrieval was performed by boiling for 15 min in a microwave oven in 10 mM citric acid (pH 6.0). The sections were blocked with 5% BSA (Solarbio; Beijing, China) at room temperature for 60 min and incubated with primary anti-RUNX2 antibody and p-Akt antibody at room temperature for 2 h. After washing three times in PBS, the sections were incubated with biotinylated anti-rabbit secondary antibodies (Boster, China) in PBS for 30 min at 37 °C, incubated with avidin-biotin-peroxidase solution (SABC kit, Boster, China), and visualized with a DAB kit (Boster, China). Immunoreactivity was visualized with a 3,3’-diaminobenzidine tetrahydrochloride kit (ZSGB-BIO, Beijing, China).

### Statistical analyses

Each group was analyzed in triplicate. We conducted statistical analysis using SPSS 20.0 (SPSS, Chicago, IL). The data are presented as the mean ± SD. Differences among multiple groups were analyzed using one-way analysis of variance (ANOVA) followed by Dunnett’s multiple comparison post hoc test. *P*< 0.05 was considered statistically significant.

## Results

### Curculigoside effectively promotes the proliferation of ADSCs

**Fig. 1:**
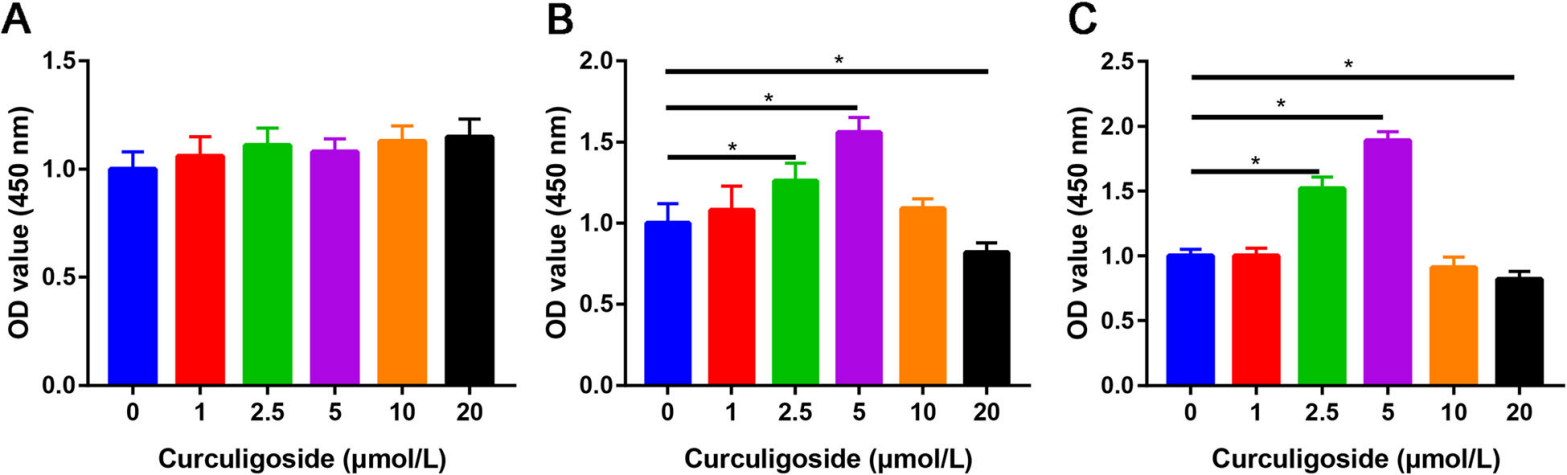
The effects of curculigoside on ADSC growth at 24, 48, and 72 h were assessed by CCK-8 assay. CCK-8 assay of cell proliferation in ADSCs after treatment with different concentrations of curculigoside at 24 h (**a**), 48 h (**b**), and 72 h (**c**)

The CCK-8 results indicated that there were no significant differences between the groups treated different concentrations of curculigoside for 24 h (Fig. 1a). The CCK-8 assay indicated that curculigoside did not induce cytotoxicity at a concentration of 5 μmol/L at 48 h (Fig. 1b). The CCK-8 results at 72 h were consistent with the CCK-8 result at 48 h (Fig. 1c). We therefore chose 5 μmol/L curculigoside as an optimal dose for further investigations.

### Bioinformatic analysis of curculigoside

**Fig. 2:**
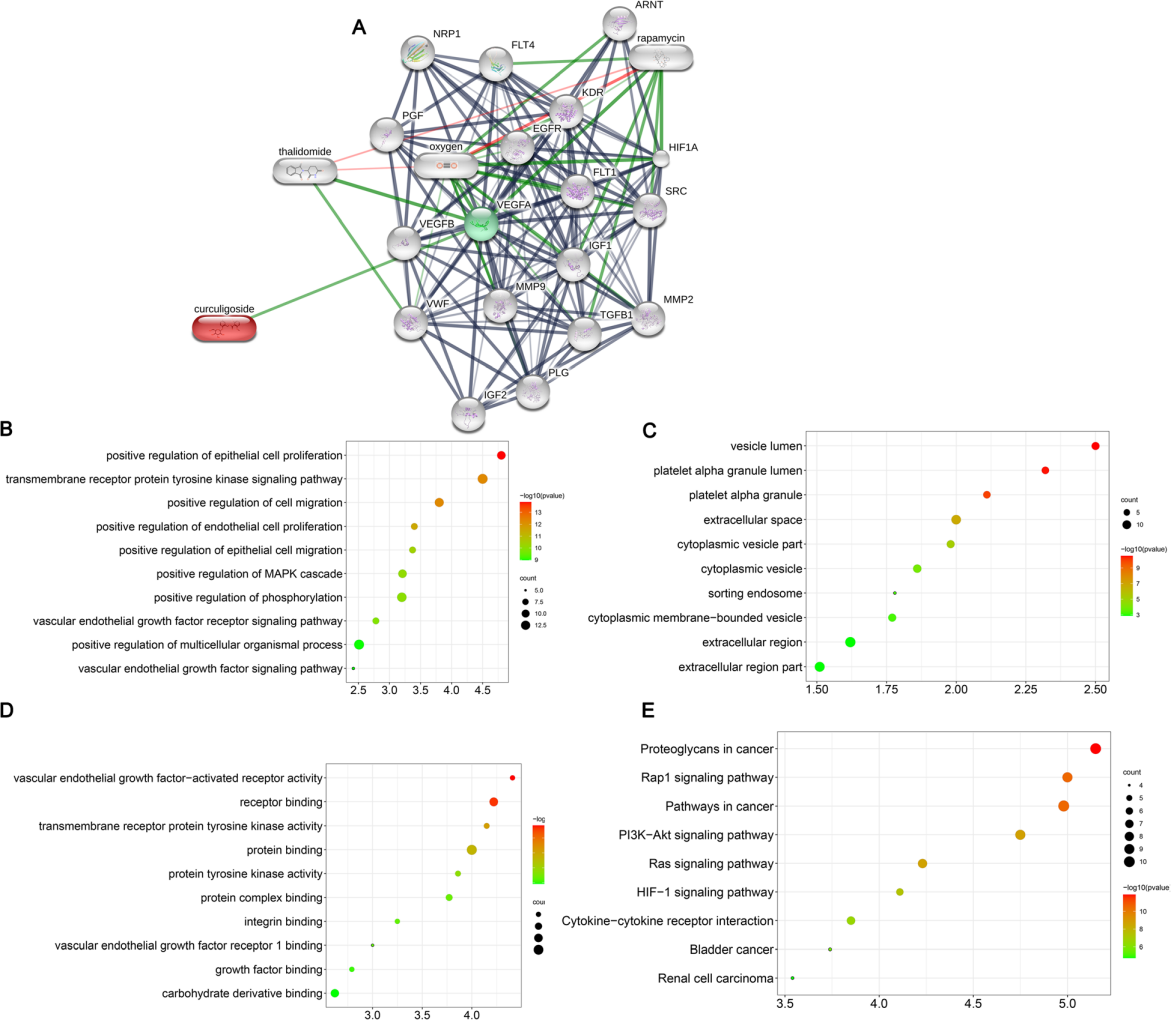
Bioinformatic analysis of curculigoside through the STITCH database. **a** Compound-gene interaction network. **b** Biological process of the curculigoside target genes. **c** Cellular component of the curculigoside target genes. **d** Molecular function of the curculigoside target genes. **e** KEGG pathway analysis of the curculigoside target genes

To demonstrate the function of curculigoside, we constructed a compound-gene interaction network using the STITCH database (http://stitch.embl.de/). The analysis of the data from the STITCH database indicated that VEGFB and VEGFA were the target genes of curculigoside (Fig. 2a).

The biological process of target genes of curculigoside is displayed in Fig. 2b, which indicates that the target genes of curculigoside were mainly enriched in positive regulation of epithelial cell proliferation, transmembrane receptor protein tyrosine kinase signaling pathway, positive regulation of cell migration, positive regulation of endothelial cell proliferation, positive regulation of epithelial cell migration, positive regulation of MAPK cascade, positive regulation of phosphorylation, vascular endothelial growth factor receptor signaling pathway, positive regulation of multicellular organismal process, and vascular endothelial growth factor signaling pathway.

The cellular components of the target genes of curculigoside are shown in Fig. 2c, which indicates that the target genes of curculigoside were mainly enriched in vesicle lumen, platelet alpha granule lumen, platelet alpha granule, extracellular space, cytoplasmic vesicle part, cytoplasmic vesicle, sorting endosome, cytoplasmic membrane-bounded vesicle, extracellular region, and extracellular region part.

The molecular functions of the target genes of curculigoside are shown in Fig. 2d, which indicates that the target genes of curculigoside were mainly enriched in vascular endothelial growth factor-activated receptor activity, receptor binding, transmembrane receptor protein tyrosine kinase activity, protein binding, protein tyrosine kinase activity, protein complex binding, integrin binding, vascular endothelial growth factor receptor 1 binding, growth factor binding, and carbohydrate derivative binding.

KEGG enrichment analysis indicated that the target genes of curculigoside were mainly involved in proteoglycans in cancer, Rap1 signaling pathway, pathways in cancer, PI3K-Akt signaling pathway, Ras signaling pathway, HIF-1 signaling pathway, cytokine-cytokine receptor interaction, bladder cancer, and renal cell carcinoma (Fig. 2e).

### Curculigoside effectively promotes osteogenic differentiation of ADSCs

**Fig. 3:**
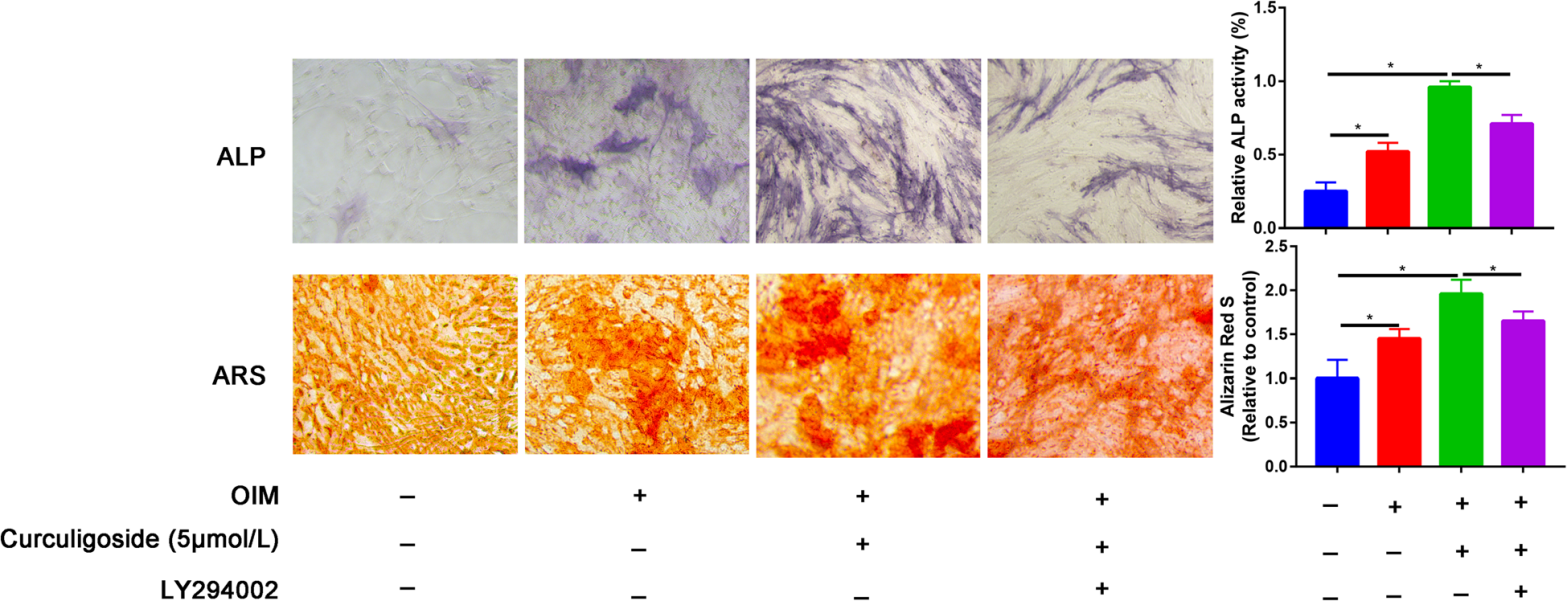
ALP activity on day 7 and ARS mineralization on day 14 after osteogenic induction in different treatment groups (control, OIM, OIM+ curculigoside, OIM+ curculigoside+LY294002)

To explore the function of curculigoside on the osteogenic differentiation of ADSCs, we performed an ALP activity assay and AR staining. The ALP and ARS results revealed that the induced group had higher ALP activity and calcium deposition than the control group. Moreover, curculigoside promoted biomineralization along with ALP activity and ARS staining compared to the induced and control groups, and these effects were partially inhibited by LY294002 (Fig. 3).

### Curculigoside effectively promotes osteogenic differentiation of ADSCs

**Fig. 4:**
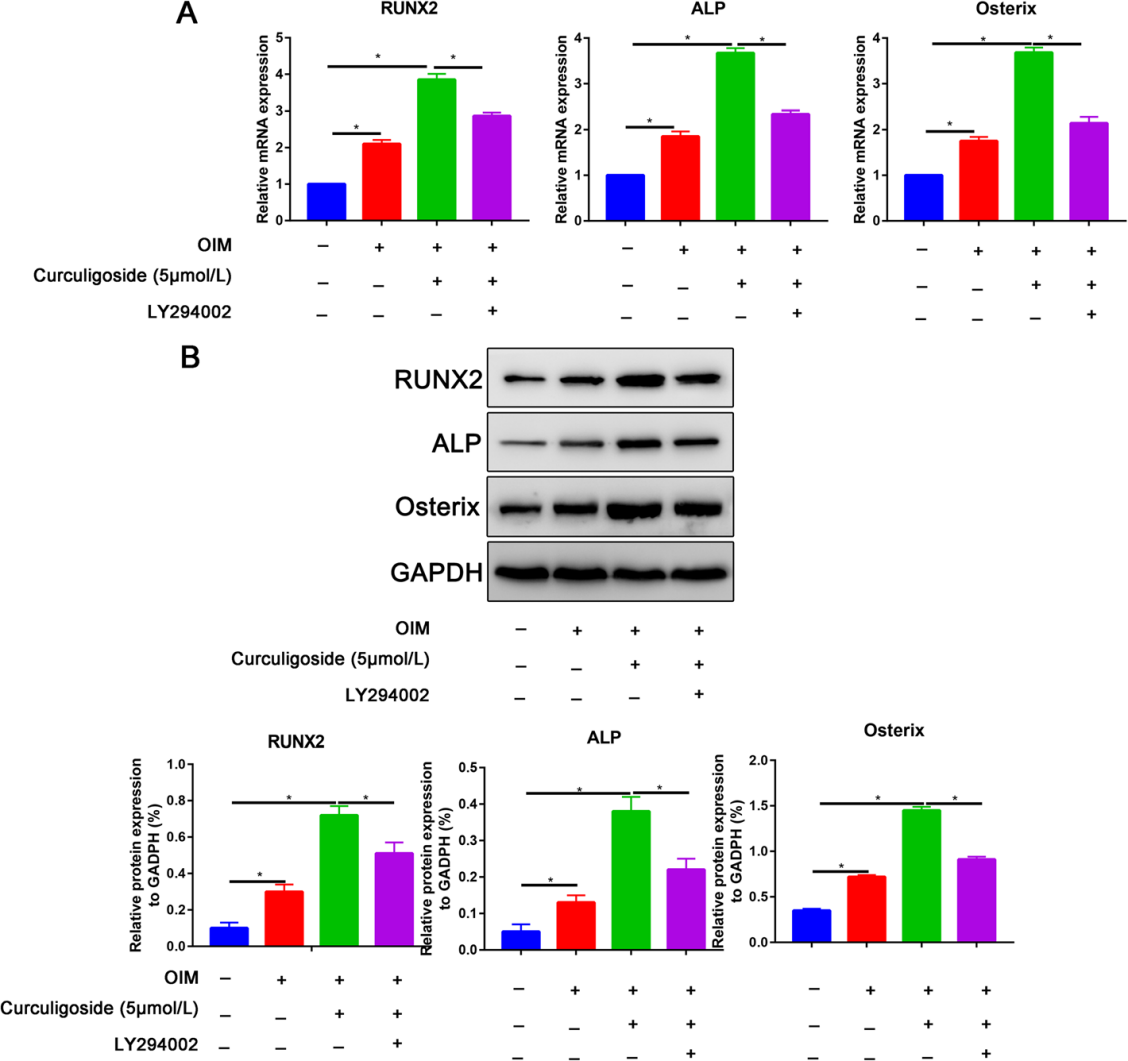
Curculigoside promoted osteogenic-related marker expression. **a** Expression levels of osteogenic gene markers, including RUNX2, ALP, and Osterix, as determined by qRT-PCR at 7 days of osteogenic differentiation. **b** Expression levels of osteogenic gene markers, including RUNX2, ALP, and Osterix, as determined by western blot assay at 7 days of osteogenic differentiation

The RT-PCR results indicated that osteogenic induction significantly increased RUNX2, ALP, and Osterix expression (Fig. 4a). The curculigoside group also exhibited significantly increased RUNX2, ALP, and Osterix expression compared with the osteogenic induction group (Fig. 4a), whereas these promotion effects were partially reversed by the PI3K inhibitor LY294002 (Fig. 4a). The western blot analysis results were consistent with the qRT-PCR results, showing that the protein expression of RUNX2, ALP, and Osterix was upregulated in curculigoside-treated ADSCs but was partially reversed by the PI3K inhibitor LY294002 (Fig. 4b).

### PI3K/Akt signaling is activated by curculigoside-treated ADSCs

**Fig. 5:**
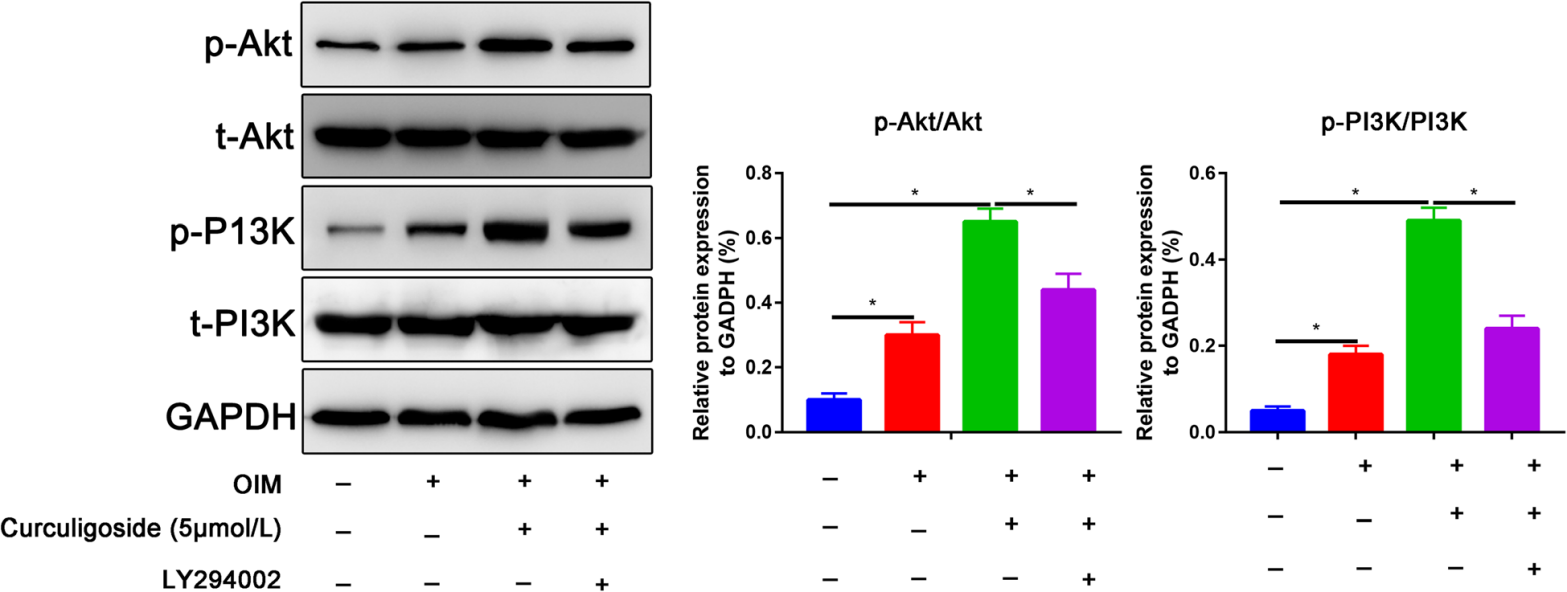
Expression levels of PI3K/Akt signaling pathway markers, including p-PI3K and p-Akt, at 7 days of osteogenic differentiation as determined by western blot assay

The phosphorylation of AKT to P-AKT is central to the PI3K/Akt pathway, and its activation stimulates the osteogenic differentiation of ADSCs. As shown in Fig. 5, phosphorylation of AKT to P-AKT was increased after osteogenic induction of ADSCs. The curculigoside group also exhibited significantly increased phosphorylation of AKT to P-AKT compared with the osteogenic induction group (Fig. 5), whereas the phosphorylation of AKT to P-AKT was partially reversed by the PI3K inhibitor LY294002.

### Curculigoside prevented bone mass loss in the OVX mouse model

**Fig. 6:**
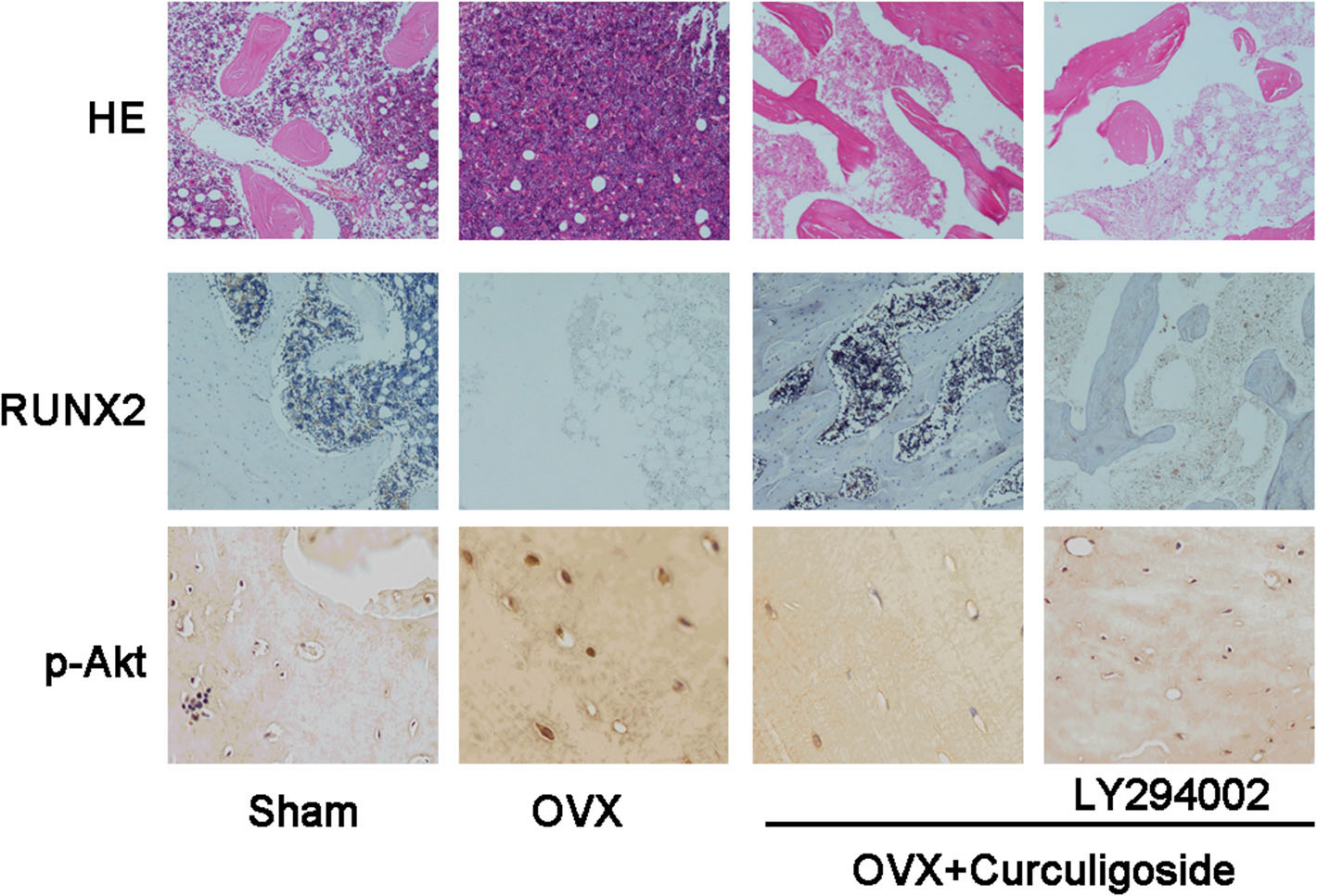
HE staining and immunohistochemical staining of RUNX2 and p-Akt in the sham, OVX, OVX+ curculigoside, and OVX+ curculigoside+LY294002 groups

HE staining showed that the trabecular bone was sparse and reduced in the OVX model group compared to the sham group. However, after treatment with curculigoside, the mice had a higher bone volume than the OVX mice, suggesting partial protection from cancellous bone loss. In addition, when LY294002 was preadded, the protective effects of curculigoside could be neutralized (Fig. 6).

Immunofluorescence staining found significantly downregulated expression of RUNX2 in the OVX mice compared with the sham mice. However, after treatment with curculigoside, the mice had a higher expression of RUNX2, suggesting that curculigoside could enhance RUNX2 expression and protect against OVX-induced bone loss (Fig. 6).

Additionally, p-AKT expression in osteoblasts was lower in OVX mice, while after treatment with curculigoside, p-Akt expression was partially elevated. In addition, when LY294002 was added, p-Akt expression was partially neutralized (Fig. 6).

## Discussion

In this study, we found that curculigoside did not induce cytotoxicity at a concentration of 5 μmol/L at 48 h. We then verified that curculigoside can promote the osteogenic differentiation of ADSCs through the PI3K/Akt signaling pathway in vitro and in vivo.

In this study, we first identified the optimal dose of curculigoside for ADSC proliferation. We measured ADSC viability for up to 72 h and found that the optimal dose of curculigoside was 5 μmol/L. Then, ALP and ARS staining were performed to assess the osteogenic effect of curculigoside on ADSCs. The results suggested that curculigoside plays a positive role in promoting the osteogenic differentiation of ADSCs. To further explore the mechanism underlying the effect of curculigoside in the osteogenic differentiation of ADSCs, the mechanism associated with curculigoside was further analyzed using the STITCH database to explore chemical-protein interactions. The STITCH database was utilized for the construction of compound-target and compound-target pathways. PI3K/Akt was the most significantly enriched pathway. Zhang et al. [26] found that curculigoside protects against excess iron-induced bone loss by attenuating Akt-FoxO1-dependent oxidative damage in mice and osteoblastic MC3T3-E1 cells. Curculigoside, one of the main bioactive phenolic compounds isolated from the rhizome of *Curculigo orchioides* Gaertn, is reported to have potent antioxidant and anti-osteoporotic properties. However, whether curculigoside plays a beneficial role in promoting osteogenic differentiation of ADSCs is unknown.

The PI3K-AKT signaling pathway is activated in the osteogenic differentiation of multiple stem cells, including ADSCs [27]. The PI3K/AKT signaling pathway has been shown to be critical for all phases of osteoblast differentiation and maturation, bone development, and growth [28]. Blocking the PI3K/AKT signaling pathway has also been shown to impair longitudinal bone growth and to prevent osteoblast differentiation [29]. In this study, we also found that p-PI3K and p-AKT expression was increased in ADSCs undergoing osteogenic induction and in curculigoside-treated ADSCs. Blocking the PI3K/Akt signaling pathway partially reversed the promoting effects of curculigoside on the osteogenic differentiation of ADSCs. Han et al. [15] predicted targets of curculigoside A in osteoporosis and rheumatoid arthritis using network pharmacology and experimental verification. The results showed that curculigoside A mainly targeted the EGFR, MAP2K1, MMP2, FGFR1, and MCL1 genes. These genes were involved in one critical signaling pathway, namely, the PI3K/Akt pathway.

Our study has numerous limitations. First, which receptor or protein is associated with the effect of curculigoside in ADSCs is unknown, and thus, more studies are needed. Second, the use of animal models seems to be necessary to evaluate the safety of curculigoside in vivo. The optimal dose of curculigoside for the treatment of osteoporosis in a large animal model is unknown. Hence, the most suitable dose needs to be verified by further prospective large-scale studies.

## In conclusion

Our data indicated that curculigoside induced the osteogenic differentiation and mineralization of ADSCs by activating the PI3K/Akt pathway in vitro and protected against OVX-induced bone loss in vivo. Curculigoside may be a potential candidate for the pharmacological treatment of osteoporosis.

## Data Availability

All the data pertaining to the present study have been included in figure in the manuscript, and the authors are willing to share the raw data upon reasonable request.

## Abbreviations

ALP: Alkaline phosphatase
ARS: Alizarin red S
STITCH: Search tool for interactions of chemicals
ADSCs: Adipose-derived stem cells
OVX: Ovariectomized
FDA: Food and Drug Administration
PBS: Phosphate-buffered saline
OD: Optical density
PVDF: Polyvinylidene difluoride
ECL: Electrochemiluminescence
DMSO: Dimethylsulfoxide
